# Small Fiber Neuropathy: Clinicopathological Correlations

**DOI:** 10.1155/2020/8796519

**Published:** 2020-01-02

**Authors:** Endre Pál, Krisztina Fülöp, Péter Tóth, Gabriella Deli, Zoltán Pfund, József Janszky, Sámuel Komoly

**Affiliations:** ^1^Department of Neurology, University of Pécs, Medical School, Pécs, Hungary; ^2^Department of Pathology, Neuropathology Unit, University of Pécs, Medical School, Pécs, Hungary

## Abstract

Small fiber neuropathy develops due to the selective damage of the thin fibers of peripheral nerves. Many common diseases can cause this condition, including diabetes, infections, autoimmune and endocrine disorders, but it can occur due to genetic alterations, as well. Eighty-five skin biopsy-proven small-fiber neuropathy cases were analyzed. Forty-one (48%) cases were idiopathic; among secondary types, hypothyreosis (9.4%), diabetes mellitus (7%), cryoglobulinemia (7%), monoclonal gammopathy with unproved significance (4.7%), Sjögren's disease (3%), and paraneoplastic neuropathy (3%) were the most common causes. Two-thirds (68%) of the patients were female, and the secondary type started 8 years later than the idiopathic one. In a vast majority of the cases (85%), the distribution followed a length-dependent pattern. Intraepidermal fiber density was comparable in idiopathic and secondary forms. Of note, we found significantly more severe pathology in men and in diabetes. Weak correlation was found between patient-reported measures and pathology, as well as with neuropathic pain-related scores. Our study confirmed the significance of small fiber damage-caused neuropathic symptoms in many clinical conditions, the gender differences in clinical settings, and pathological alterations, as well as the presence of severe small fiber pathology in diabetes mellitus, one of the most common causes of peripheral neuropathy.

## 1. Introduction

The majority of cases with peripheral neuropathy has a combined involvement of large and small nerve fibers, but sometimes, the damage of different types of fibers are unequal. Certain diseases cause predominantly large fiber damage (e.g., B12 vitamin deficiency), others prefer a small fiber lesion (e.g., Fabry's disease). Furthermore, special structures, such as axons and myelin, are usually differently involved [1].

Small fiber neuropathy (SFN) develops due to the lesion of peripheral nerve fibers with a thin myelin sheath (A*δ*) and without myelin (C fibers). These fibers are responsible for the mediation of temperature and pain sensations, as well as the control of autonomic functions; they build up to 80-90% of the peripheral nerves [2–4].

Patients suffering from SFN usually develop somatic symptoms, but autonomic dysfunctions might occur as well. Somatic symptoms can include numbness, paraesthesia, hypo- or hyperalgesia, allodynia, and neuropathic pain. Neuropathic pain is debilitating; it is characterized by burning, prickling, itching, stabbing, and “lightning-like” sensations; therefore, it has a considerable impact on quality of life [5]. Autonomic disturbances include dry eyes and mouth, abnormal sweating, altered gastrointestinal motility and bladder control, abnormal heart-rate variability, and orthostatic issues such as hypotension and tachycardia [5, 6]. Recently, a subclassification was suggested according to the dominant symptoms [7].

The frequency of SFN is not exactly known. A recent Dutch study showed an incidence rate of 11.7/100,000 and a prevalence rate of 52.9/100,000 [8].

SFN might be idiopathic, when the underlying cause cannot be identified, but several common diseases might cause it; therefore, patients with SFN have to undergo many diagnostic tests to identify or exclude metabolic, malignant, infectious, or genetic diseases [9, 10]. A further difficulty is that SFN might be an initial phase of neuropathy, and it can later progress to thick-fiber involvement as well. Further studies with long-term follow-up are required to characterize the natural evolution of SFN [11].

## 2. Materials and Methods

We performed a cross-sectional, single-institution, prospective study including a cohort of patients investigated with SFN between the years of 2012 and 2018 at the Neurology Department, University of Pécs, Medical School, Pécs, Hungary. All patients provided written informed consent before enrollment, and the study was approved by the institutional Review Board of University of Pécs, Hungary.

The inclusion criteria were as follows: (1) typical complaints related to small-fiber involvement, such as neuropathic pain; (2) physical signs of SFN, including loss of pain and/or temperature sensation, and/or autonomic signs, hyperalgesia, and allodynia, and (3) abnormal skin biopsy findings with reduced intraepidermal nerve fiber density (IENFD). According to the diagnostic criteria, all of our patients belonged to the definite SFN category [12].

### 2.1. Skin Biopsy

All patients underwent skin biopsy. The biopsy was performed according to a standardized technique. Briefly, skin biopsy specimens were obtained using a 3 or 4 mm punch from the leg, 10 cm above the lateral malleolus in local anesthesia. The samples were fixed in 4% paraformaldehyde for 24-48 hours, cryoprotected in 20% sucrose phosphate-buffered saline for 24 hours, and frozen to -80°C embedded into OCT freezing compound overnight. Fifty-micrometer-thick cryostat-cut frozen slides were used when proceeding to immunohistochemistry. After blocking with 5% bovine serum albumin, 1% lysine and 5% goat serum immunostaining of axons was performed against the panaxonal marker, PGP 9.5, with a polyclonal rabbit anti-human PGP 9.5 antibody (DAKO, Z511601-2, in a dilution of 1 : 1000 in 4°C). After a 48- to 72-hour incubation with a primary antibody, further steps with a biotinylated secondary antibody and development were carried out with the VECTASTAIN Elite ABC HRP Kit and the Vector SG substrate, respectively (Vector Laboratories). Those fibers which crossed the dermal/epidermal border were counted. The subepidermal network and the autonomic fibers supplying the sweat glands were also assessed. The integrity of the specimen was judged before the immunohistochemical procedure on a hematoxylin-eosin-stained routine slide. A minimum of 5 sections of a specimen were evaluated and averaged. Results were expressed as the number of IENF/mm according to the EFNS guidelines [13]. Values below the 0.05 quantile per age span for females and males were considered pathological as recommended [10, 14]. Subepidermal nerve fiber density (SENFD) and autonomic fiber density (ANFD) around sweat glands were semiquantitatively evaluated on all slides from all cases with a 3-grade system: 0=no fibers; 1=moderate amount of fibers; and 2=abundant fibers. In each case, the result of the best specimen was recorded, but generally, no remarkable differences were found among slides prepared from one subject.

### 2.2. Clinical Test

Detailed neurological physical examination was performed in each case, including sensory tests for tactile stimuli (monofilament), pain (pinprick), temperature (standardized temperatures), joint position sensation, vibration (tuning fork), and recording of allodynia and hyperalgesia.

All patients underwent extensive laboratory testing to exclude or prove the underlying cause, such as diabetes mellitus, renal and hepatic dysfunction, hypothyroidism, infections (hepatitis B and C and Lyme disease), autoimmune disease (immune serology for Sjögren's syndrome, systemic lupus erythematodes, rheumatoid arthritis, and vasculitis), paraproteinemia (serum electrophoresis), paraneoplastic syndromes (onconeural antibodies, chest X-ray, or CT, abdominal ultrasonography, or CT), and vitamin B12 deficiency. The patients' alcohol abuse and family history of SFN were also recorded. A blood spot test was applied for Fabry's disease.

All patients underwent detailed electrophysiology such as sensory and motor nerve conduction studies of the upper and lower extremities and electromyography of deltoid, abductor pollicis brevis, and anterior tibial muscles.

According to the results, patients were classified as (1) idiopathic SFN (iSFN, when the underlying cause was not found, electrophysiology was negative, and IEFD was decreased); (2) secondary, pure SFN (sSFN, when the underlying cause was identified, electrophysiology was negative, and IEFD was decreased); (3) SFN with axonal neuropathy; and (4) SFN with demyelinating neuropathy (regardless of the underlying cause, but with decreased IEFD and positive electrophysiology). A detailed analysis was only performed for the isolated SFN groups (1 and 2).

### 2.3. SFN-Related Tests

The Toronto clinical neuropathy scoring system (TCNS) was recorded for each case to assess the severity of neuropathy. It is a weighted scoring system for symptoms of neuropathic pain, sensory loss, motor functions, and deep tendon reflexes of the lower limb; therefore, large and small fiber functions are included as well [15]. The Douleur neuropathique 4 questionnaire (DN4) was applied for the screening of neuropathic pain (NP) [16]. The Pain Detect Questionnaire (PD-Q9) and The Neuropathic Pain Scale (NPS) were used to evaluate different pain qualities associated to NP [17, 18]. Both are simple, self-administered tests, allowing the detection dimensions and different qualities of NP on a quantitative scale [19]. A Hungarian form of the Beck Depression Inventory (BDI) was administered for the assessment of depression [20]. Finally, the pain intensity was recorded on an 11-point visual analogue scale (VAS).

### 2.4. Statistics

Differences were compared by Student's *t*-test for continuous variables and by chi-square test or ANOVA for categorical variables. Normality test was performed for all continuous variables. The data analysis was performed using the SPSS v.25 statistical program (IBM Inc., Chicago, USA). The level of significance was set as 0.05.

## 3. Results

Between the years of 2012 and 2018, we found 117 patients fulfilling the criteria of biopsy-proven small-fiber involvement. Eighty-five of them were pure SFN (35% idiopathic, 37.6% secondary), 23 patients (19.7%) had SFN associated with axonal neuropathy, and 9 patients (7.7%) with demyelinating large fiber neuropathy. For further analysis, we included only the isolated SFN patients. Forty-one patients (48%) of the pure SFN group were idiopathic.  Table 1 shows the comparison of the basic characteristics of patients with iSFN or sSFN. Two thirds (68%) of the study population were female, and this predominance was even significantly higher in the sSFN group. The disease started 8 years later in the sSFN (*p* < 0.05). The distribution of clinical symptoms followed a length-dependent pattern in the vast majority of the cases (85%), and only occasional patients were found with burning mouth and vulvodynia or with diffuse complaints. Typical complaints of neuropathic pain were found, but the quantitative evaluation was limited because almost all patients were under treatment.

The results of NP-related scoring are presented in Table 2. All recorded parameters were in the middle range, including VAS, DN4, PD-Q9, and NPS. DN4, PD-Q9, and NPS were positive in 68, 81, and 42%, respectively. TCNS results were in the lower middle range, because it measures small- and large-fiber involvement as well. It showed a mild, moderate, or severe neuropathy in 19, 10, and 2%, respectively. BDI was normal in the majority of the cases, and a mild to moderate depression was only detected in 20% of the iSFN group.

**Figure 1:**
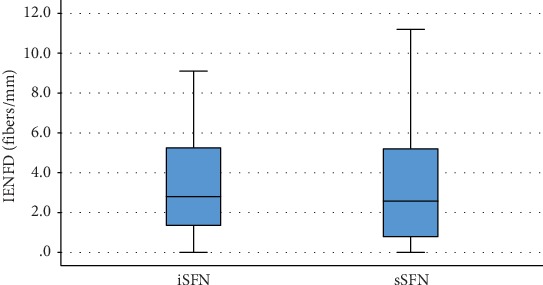
Intraepidermal nerve fiber density (IENFD) in idiopathic (iSFN) and secondary SFN (sSFN). No significant differences were found between the two cohorts.

IENFD was 3.2 ± 2.7 fibers/mm (mean ± SD), but it varied in a large scale from 0 to 11.  Figure 1 demonstrates that the results were comparable in idiopathic and secondary SFN patients, but the distribution did not follow the normal pattern (not shown).

The analysis of the subgroups showed more severe small fiber loss in men compared to women (IENFD was 2.34 ± 1.97 fibers/mm and 3.6 ± 2.94 fibers/mm, respectively, *p* < 0.05). Patients with diabetes had lower IENFD compared to nondiabetic patients (IENFD was 0.79 ± 0.58 fibers/mm and 3.4 ± 2.75 fibers/mm, respectively, *p* < 0.05). Compared to those patients whose IENFD was below or above 5 fibers/mm, we found that DN4 was significantly higher (5.5 ± 2.99 and 4.74 ± 1.94, respectively, *p* < 0.05) and patients were more depressed, as BDI showed (8.0 ± 7.5 and 3.5 ± 2.88, respectively, *p* < 0.05) in the group with more severe pathology.

**Figure 2:**
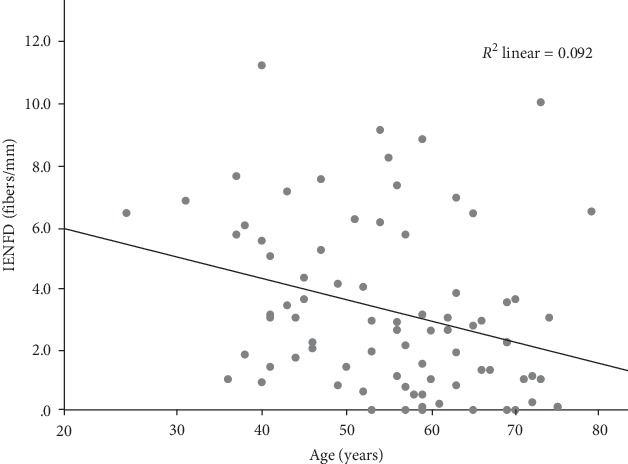
Relationship of the IENFD and age. Intraepidermal nerve fiber density (IENFD) showed negative correlation with the age of the investigated subjects.

IENFD showed significant negative correlation with the age of patients (*r* = −0.304, *p* < 0.01) (Figure 2).

Subepidermal nerve fiber density was variable, but it was usually comparable to IENFD. Grades 0, 1, and 2 were found in 29%, 59%, and 12%, respectively; therefore, the majority of the cases presented moderate fiber loss. In opposite, the autonomic innervation was usually spared (cases with grade 0, 1, and 2 were 15%, 36%, and 49%, respectively).

**Figure 3:**
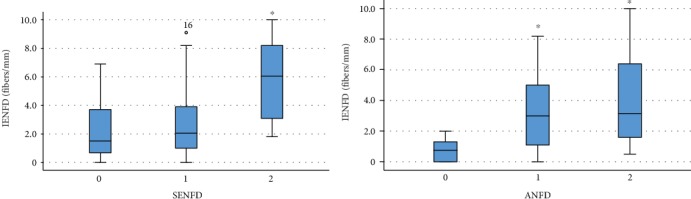
Correlations of intraepidermal, subepidermal, and autonomic fiber densities. Although, subepidermal nerve fiber density (SENFD) and autonomic nerve fiber density (ANFD) were assessed semiquantitatively, the amount of these fibers was comparable to intraepidermal nerve fiber density (IENFD). Asterisks mark significant differences from grade 0.

Statistical analysis resulted in significant association between IENFD, SENFD, and ANFD, but it was absent when the histological findings were compared to clinical variables. Generally, low SENFD and ANFD were associated with low IENFD. Significant differences in IENFD were found between grade 0 and grade 2 of SENFD (*p* < 0.05), and it was also significant when we compared grade 0 to grade 1 or 2 of ANFD (*p* = 0.01 and *p* < 0.01, respectively) (Figure 3).

The most common causes of sSFN were hypothyroidism (Hashimoto's disease), diabetes mellitus, and cryoglobulinemia. Monoclonal gammopathy of undetermined significance (MGUS), Sjögren's syndrome, and paraneoplastic process were rare, as well as Lyme disease (Table 3). Among the remaining secondary cases, routine laboratory tests resulted in renal dysfunction (1 case) and antinuclear antibody positivity without systemic autoimmune symptoms (3 cases). Vitamin B12 levels, viral serology, and the Fabry tests were all normal.

Pain-killing medication was administered to 64 patients (75%), and half of them received combined treatment. The most common medications were benzodiazepines (32%), tricyclic antidepressants (23%), serotonin-norepinephrine reuptake inhibitors (17%), gabapentin (17%), pregabalin (15%), and tramadol-opioids (14%). Four patients received immune modulatory treatments.

We found significant gender differences in pain scores. Mean of DN4, PD-Q9, NPS, and VAS was significantly higher in cases of female patients compared to males (all *p* < 0.05).

Although the minority of patients had depression, a significant correlation was found among BDI score and VAS or PD-Q9 (*r* = 0.659, *p* < 0.05 and *r* = 0.818, *p* < 0.05, respectively).

## 4. Discussion

The clinical presentation of SFN is heterogeneous, and the most frequent pattern is a length-dependent polyneuropathy, characterized by the typical symptoms appearing on the distal part of the extremities, mostly on feet; rarely, a non-length-dependent neuropathy can appear, mainly with patchy symptoms in a certain part of the body, such as the face, tongue, and trunk, as well as multiple mononeuropathy [6, 21]. In our cohort, the non-length-dependent SFN occurred in 15%, according to the clinical findings. We did not find differences in either IENFD or other clinical data, regarding the distribution. In opposite, Khan and Zhou reported a lower frequency of diabetes mellitus and a higher frequency of autoimmune diseases in the non-length-dependent group. The ratio of females was higher, and the onset was earlier among those patients [21].

The diagnosis of SFN is still challenging despite of increasing knowledge and available diagnostic tools. Clinical criteria were established only for the length-dependent form; in other cases, the diagnosis is more difficult. The assessment of the IENFD is a noninvasive and sensitive method to prove the disease; it was recommended by the European Federation of Neurological Societies (EFNS) and the Peripheral Nerve Society (PNS) in 2010 with level A evidence. Additionally, the assessment of intraepidermal nerves results not only in quantitative measures but prognostically important morphological changes can also be observed, such as length, branching, and axonal swelling [13, 22, 23]. Here, we did not assess morphological changes other than the count of intraepidermal fibers, because this parameter was accepted as the evidence of SFN.

Generally, alternative assessments for evidence of SFN quantitative sensory testing (QST) [24] and contact heat-evoked potential test (CHEP) are recommended; however, the first one is time consuming and contains subjective domains, and the latter is not widely available [24, 25].

Based on the extensive investigations, less than half of the patients were classified as idiopathic SFN in our study. This is slightly lower than was reported in previous publications (53-76%) [9, 21, 26–28]. One possible explanation is that many patients had hypothyreosis in our cohort. In these cases, the causality was not proven, but they were classified as sSFN. Mild gender differences were common in previous studies with a ratio of females between 41 and 58% [21, 26, 28], but it was 71% in a study [27]. Our cohort was similar to the latest one with 68% female predominance.

A battery of neuropathy tests was used, because physical examination was reported having a low diagnostic accuracy [28]. Variable results were published about correlations of physical alterations, neuropathy scores, and IENFD. Loss of pain sensation and pain intensity on VAS were reported to be related to IENFD [29, 30]. In our study, the DN4 score was the only finding that was significantly related to the severity of the intraepidermal fiber loss.

Comparing iSFN and sSFN, significant differences were found in the ratio of genders, age of the patients, and disease onset. None of the remaining investigated parameters was significantly different between the above groups, including distribution of symptoms and types of pain qualities, as well as pain intensity and results of neuropathy scoring. These data might indicate that loss of intradermal thin fibers results to similar clinical symptoms regardless of the underlying causes.

We found a significant effect of gender on IENFD, but it was not related to the age and the type of lesion, as well as the etiology. Interestingly, higher IENFD (less severe pathology) and higher pain scores (more severe clinical appearance) were found in female patients, but close correlation was not found between them, with the exception of DN4 score. In previous studies, variable gender effects have been reported. The gender difference in IENFD in a healthy population is well known, and it seems the pathology follows this trend.

The second important finding of our study is the effect of diabetes on SFN. Diabetes-induced SFN has earlier been found to be associated with more severe pathological changes [28, 31, 32], which we confirmed here. Although TCNS was reported with the highest diagnostic yield in diabetic neuropathy [33], here we found positive results in only 30.7% of the cases, which can be explained by the absence of large-fiber involvement. Recently, corneal confocal microscopy (CCM) has been proven to be a sensitive and comparable method to skin biopsy in the diagnostics of diabetic SFN [24, 34]. Further studies in large cohorts of SFN with a different etiology are necessary to confirm the reliability of CCM as a diagnostic tool in SFN and its comparison to histological methods. Because of the limited availability of pain-related evoked potential tests and CCM, QST and skin biopsy remain the standard diagnostic procedures in case of SFN. Precise procedure and strict usage of normal values are necessary for reliable results.

A limited number of studies investigated SENFD and ANFD, and no clear clinical importance of their changes was determined. Furthermore, less clear-cut diagnostic criteria were established for these pathological changes, and the quantification is more difficult. Although in our study low IENFD was statistically associated with low SENFD and ANFD, in our practice, the autonomic innervation of sweat glands has remained intact or minimally involved even in severe SFN cases, and therefore, staining of nerve fibers around sweat glands might serve as a quality control of immunohistochemistry.

The therapy of our patients was conducted according to the guidelines of neuropathic pain treatment [35], but, somehow, benzodiazepine usage was common. It can be explained by the anxiety of patients due to the sort of investigations and the chronic troublesome pain.

Our study was limited because we applied only a cross-sectional investigation, and it is known that IENFD may change in time and due to clinical conditions; therefore, a long-term follow-up study would be recommended. Furthermore, no additional clinical tests, such as QST or CCM were systemically carried out for comparison because of limited time and availability of the tools. The pain intensity assessment was also limited, because the majority of patients was on pain medication. A genetic survey was not conducted, either.

In summary, our results are in line with previous publications. We found significant differences of IENFD in SFN regarding gender and the presence of diabetes. Although, the frequency of SFN is nor clearly known, it can be variable according to race and gender. The number of possible underlying conditions is significant, and we have to perform all recommended tests to exclude the potentially treatable forms, otherwise only symptomatic therapy is available for patients.

## Figures and Tables

**Table 1:** Basic characteristics of the study population.

	iSFN (*n* = 41)	sSFN (*n* = 44)	Sign.
Sex (female)	26 (41%)	32 (73%)	*p* < 0.05
Age (ys)	51.4 ± 12.5	58.7 ± 10.9	*p* = 0.05
Onset (ys)	47.6 ± 12.6	55.6 ± 11.1	*p* < 0.05
Duration (ys)	3.9 ± 3.0	3.2 ± 2.9	ns.
Distribution, LD/NLD (*n*, %)	35/6 (85/15%)	37/7 (84/16%)	ns.
Upper extremity involvement (*n*, %)	26 (63%)	26 (59%)	ns.
Numbness (*n*, %)	34 (83%)	35 (80%)	ns.
Burning pain (*n*, %)	26 (63%)	25 (57%)	ns.
Prickling pain (*n*, %)	12 (29%)	11 (25%)	ns.
Itching pain (*n*, %)	5 (12%)	3 (7%)	ns.
Allodynia (*n*, %)	10 (24%)	10 (23%)	ns.

Although the ratio of females and the onset of the disease was significantly higher in the secondary SFN (sSFN) group, all other parameters were not statistically different from idiopathic SFN (iSFN). LD: length dependent; NLD: nonlength-dependent; ns.: nonsignificant.

**Table 2:** The main findings of pain-related tests in the study population.

	iSFN (*n* = 41)	sSFN (*n* = 44)	Sign.
BMI (kg/m^2^)	26.0 (4.3)	27.4 (4.9)	ns.
Pain intensity (VAS)	5.5 (2.3)	6.1 (2.4)	ns.
DN4	5.0 (2.5)	4.9 (2.0)	ns.
painDetect (PD-Q9)	13.9 (7.2)	12.1 6.0)	ns.
NPS	35.0 (20.7)	42.0 (26.6)	ns.
TCNS	4.1 (2.5)	5.1 (2.7)	ns.
IENFD (fibers/mm)	3.3 (2.5)	3.1 (3.0)	ns.
SENFD	0.8 (0.6)	0.8 (0.6)	ns.
ANFD	1.3 (0.6)	1.4 (1.0)	ns.

Data represent the mean and (SD) of the investigated parameters. There were no significant differences between idiopathic (iSFN) and secondary SFN (sSFN) in respect of the majority of the investigated parameters. ANFD: autonomic nerve fiber density; IENFD: intraepidermal nerve fiber density; NPS: Neuropathic Pain Scale; SENFD: subepidermal nerve fiber density; TCNS: Toronto Clinical Neuropathy Scoring System; VAS: visual analogue scale; ns.: not significant difference.

**Table 3:** The most common diseases associated with SFN.

Disease	Frequency *N* (%)
Hashimoto	8 (9.4)
Diabetes	6 (7)
Cryoglobulinemia	6 (7)
MGUS	4 (4.7)
Sjögren's syndrome	3 (3.5)
Malignancy	3 (3.5)
Lyme disease	2 (2.3)

MGUS: monoclonal gammopathy of undetermined significance; N: number of cases.

## Data Availability

No data availability statement is included.

## Abbreviations

BDI: Beck Depression Inventory
CCM: Corneal confocal microscopy
DN4: Douleur neuropathique 4 questionnaire
IENFD: Intraepidermal nerve fiber density
NP: Neuropathic pain
NPS: Neuropathic Pain Scale
PD-Q9: Pain Detect Questionnaire
QST: Quantitative sensory testing
SFN: Small fiber neuropathy
TCNS: Toronto clinical neuropathy scoring system
VAS: Visual analogue scale.
